# Opioid Dose Reduction Vis-à-Vis Lumbar Medial Branch Intervention

**DOI:** 10.7759/cureus.84229

**Published:** 2025-05-16

**Authors:** David H Rustom, Alexander A Restum, Kevin F Henry

**Affiliations:** 1 Pain Management, Wayne State University Detroit Medical Center, Detroit, USA; 2 Medical Education, Wayne State University School of Medicine, Detroit, USA; 3 Physical Medicine &amp; Rehabilitation, Rehabilitation Institute of Michigan, Detroit Medical Center, Detroit, USA

**Keywords:** lumbar facet arthropathy, lumbar facet joint pain, lumbar medial branch block, lumbar medial branch block (lmbb), prescription opioid abuse, radiofrequency ablation (rfa), spinal intervention, spinal pain intervention

## Abstract

Introduction

Lumbar facet arthropathy (LFA) is a chronic degenerative condition that evolves over time. Osteoarthritis is defined by bony overgrowth, joint effusions, and synovial cysts. These findings are also seen in LFA, as there is degeneration of the facet joints throughout the spine. It primarily affects individuals >40 years of age and is a leading cause of low back pain. The most affected region of the lumbar spine includes the L4/L5 and L5/sacral ala junctions. We investigated the association between lumbar medial branch blocks (LMBBs) and radiofrequency ablations (RFAs), and the reduction in prescription opioid use in patients with LFA.

Methods

This is a retrospective cohort study of patients presenting to an outpatient pain medicine clinic with documented LFA. A total of 526 patients with LFA who underwent an LMBB, RFA, or a combination of both procedures were evaluated for morphine-equivalent opioid prescriptions in the outpatient clinic before and after the interventions. We used a paired t-test to compare pre- and post-procedure results to assess changes in prescribed morphine equivalent medications.

Results

There were statistically significant reductions in prescription opioid use when standardized to morphine equivalency in patients who underwent an LMBB or a combination of both LMBB and RFA procedures. For patients who underwent an LMBB procedure, the mean reduction in opioid equivalence dose from pre- to post-procedure was 4.06 mg. Among those who received both LMBB and RFA, the reduction was 6.10 mg, suggesting that the addition of RFA may further enhance the decrease in opioid use.

Conclusions

We found that individuals who underwent an LMBB or a combination of LMBB and RFA procedures had a significant reduction in morphine-equivalent drug use within the outpatient pain medicine clinic. These findings support the effectiveness of these procedures in reducing pain from LFA and highlight their potential to lessen the associated healthcare burden.

## Introduction

Lumbar facet arthropathy (LFA) is a degenerative condition affecting the cartilage of the facet joints in the lower spine, resulting in inflammation and lumbar pain. These facet joints are paired, true synovial joints that constitute the posterolateral articulations between adjacent vertebrae [1]. Anatomically, they are located between the pedicle and lamina of the same vertebral level. Each facet joint connects the vertebral arch of one vertebra to the adjacent vertebra, with a synovial membrane and articular cartilage facilitating smooth movement [2]. These lumbar facet joints can be damaged by inciting events; however, more commonly, they are damaged due to repetitive strain, obesity, and microtrauma over a lifetime. Although the etiology of facet joint damage may vary, the typical pathogenesis involves degeneration of the hyaline cartilage, progressive joint space narrowing, and sclerosis of the subchondral bone [3]. These joints are the primary stabilizers of the vertebral column and aid in movements of the trunk and pelvis, including extension, flexion, and rotation [2].

Damage to the joints leads to pain localized to the neck and lower back, with some patients reporting radiation to the upper or lower limbs [4]. This pain is transmitted via the facet joint’s dual innervation from the medial branches of the posterior primary rami at the same level and one level above [5]. Most of the facet pain originates from the L4-L5 and L5-S1 facet joints. At the L4-L5 level, the L4 nerve innervates the superior articular process of L5 and the inferior articulation of the L4-L5 facet joint. In contrast, the L5-S1 facet joint differs from other lumbar facet joints in that it is innervated by the medial branch of L4 and the dorsal ramus of L5, with the latter coursing along a groove formed between the base of the S1 superior articular process and the sacral ala [6].

Referral pain patterns involving the posterior and anterior pelvis have been infrequently described in the literature. LFA can contribute to conditions that produce radiculopathic features by compressing the neural foramen, the exit point of the lumbar spinal nerves. Diagnosis is typically based on physical examination maneuvers, with radiologic imaging used for confirmation. Pain from LFA can be provoked via lumbar extension combined with 20-degree axial rotation and downward load (facet loading sign) [3]. Patients also report localized pain over the facet joints, typically located 2-3 fingerbreadths lateral to the spinous processes. Imaging, such as computed tomography (CT) or magnetic resonance imaging (MRI), allows clinicians to assess the extent of degeneration, the degree of nerve impingement, and the impact on surrounding structures. CT findings commonly present joint space narrowing, subchondral sclerosis, and osteophyte (bone spur) formation [7]. In contrast, MRI may reveal adjacent disc changes, synovial cyst formation, degeneration of the articular cartilage, or hypertrophy of the facet joint itself [7].

Lower back pain is reported in 70-80% of the population, and the lumbar facet joint is the source of pain in 15-40% of cases [8]. Pain from LFA, along with other causes of lower back pain, often leads patients to seek relief through opioid medications. However, this reliance on opioids increases the risk of addiction and dependence. The United States is amidst an opioid epidemic, one of the largest public health crises that poses a serious threat to the well-being of Americans while placing a substantial burden on the U.S. healthcare system. The issue of opioid use and overdose has progressively worsened. In 2017, 47,600 Americans died of opioid related drug overdoses, a 12.0% increase from 2016 [9]. While access to illicit substances can be attributed to the rise of opioid related overdoses, the CDC estimates that 130 people each day die from an opioid overdose, with approximately half involving prescription opioids [10].

The epidemic has a tremendous human toll as well as an economic toll, as the U.S. healthcare system has been struggling under the burden. In 2017, costs for opioid use disorder and fatal overdoses were estimated to be 1.02 trillion when accounting for reduced quality of life and the value of life lost due to fatal opioid overdose [11]. Early in the crisis, medical expenses on the U.S. healthcare system were estimated to be $15,884 for opioid abusers and $1,830 for non-abusers [12]. As medical professionals who have the ability to prescribe opioids, it is our duty to look for interventions and strategies that can reduce our patients’ reliance on opioids. For patients who have struggled with opioid use to treat lower back pain, physicians have increasingly turned to repeated procedures, including radiofrequency ablation (RFAs) and lumbar medial branch block (LMBBs) for pain relief. Some studies have found an association between RFAs and the reduction of prescription opioid refills 180 days after each procedure [13].

The treatment of lumbar pain is extremely costly, with the facility costs for an office visit being $93.80 for each visit, representing the extreme healthcare costs associated with chronic pain management [14]. The treatment of this pain can take many forms, ranging from medication and non-invasive therapy to pain management procedures such as LMBBs or RFAs. Some research has even outlined positive long-term pain relief for patients with chronic lower back pain from LFA when they are treated with physiotherapy [15]. When non-interventional methods fail to provide adequate pain relief, an LMBB with an RFA is indicated for pain. LMBB is a diagnostic tool that uses a local anesthetic agent to block the medial branch nerve and inhibit incoming pain stimuli from the affected arthritic joints [16]. LMBBs are seen as a prognostic tool for eventual RFA. If patients respond positively to an LMBB and report a >80% reduction in pain after two procedures, they are considered to have a favorable prognosis for proceeding with lumbar RFA [17]. RFA is a procedure that uses heat to disrupt pain signals by thermally deadening the medial branch nerve fibers, thereby preventing pain transmission from the lumbar facet joints [18]. While the cartilage of the facet joint is aneural, pain from degeneration is elicited by nociceptors in the surrounding joints and facet [4].

Pain imposes a significant economic burden on individuals, the healthcare system, and society at large. The fiscal impact stems from direct healthcare costs, lost productivity, and disability-related expenses. The total economic cost of pain is estimated to range from $560 billion to $635 billion annually [19]. The American Productivity Audit found that 77% of lost productivity associated with pain was due to decreased work efficiency rather than absenteeism [11]. This highlights the substantial financial strain pain places on the country. Addressing the economic burden of pain requires better pain management strategies and responsibly administered prescriptions. There are limited studies discussing the successful reduction in opioid use as a result of these procedures. The goal of this study is to investigate the efficacy of these procedures by reviewing the opioid use of patients before and after successful completion of these interventions.

## Materials and methods

Patient selection

This retrospective cohort study was granted IRB approval through Wayne State University. A chart review helped identify 526 patients from two physician pools who had received an LMBB, RFA, or a combination of the two procedures within our testing period in our outpatient pain medicine practice. The patients were chosen in our testing period between January 2018 and January 2023. Patients who did not receive either procedure or had received an LMBB or RFA outside our testing period were excluded from the testing criteria. Patients who did not have an initial opioid prescription during the testing period were excluded from the testing criteria. All patients selected were coded and de-identified before statistical analysis.

Data gathering

**Figure 1:**
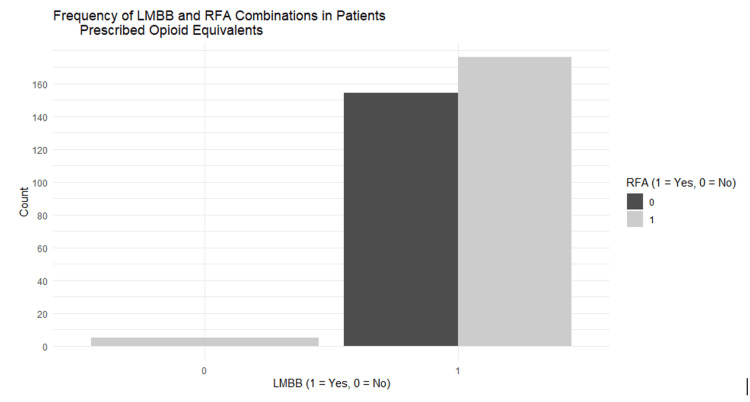
Distribution of LMBB and RFA combinations among patients prescribed opioid equivalents The bar chart illustrates the frequency of patients who received LMBB and/or RFA procedures, stratified by LMBB status (1 = received, 0 = not received) and colored by RFA status (1 = received, 0 = not received). The majority of patients who received opioids also underwent both LMBB and RFA, while very few received neither. LMBB, lumbar medial branch blocks; RFA, radiofrequency ablation

Patient panels from two independent physical medicine and rehabilitation physicians were used to gather data on the effectiveness of LMBB and RFA procedures in the reduction of pain. The prescription medications and dosages for each patient were also reviewed before and after these procedures were completed. The medications were standardized to their morphine equivalency, and the equivalencies before and after the procedures were reviewed to look for changes in the opioid use. A bar chart was created to stratify what patients received an LMBB and/or RFA to look at opioid changes based on a single procedure or the combination of both (Figure 1). These numbers were stratified to look for variations or trends in both morphine equivalency use and the number of visits to our outpatient clinic.

Statistical analysis

We initially chose the paired t-test to examine the statistical association between LMBB alone, or in combination with RFA procedures, and changes in prescription drug use, standardized to morphine equivalency, before and after the procedures. We coded the presence or absence of LMBB and RFA procedures and stratified the data to reflect changes in morphine equivalency for patients who received just LMBB, as well as those who received both LMBB and RFA procedures. The paired t-test was selected to assess the statistical association between LMBB, either alone or in combination with RFA, and changes in prescription opioid use, standardized to morphine milligram equivalents before and after the procedures. The LMBB and RFA interventions were coded as present or absent, and the data were stratified to examine changes in opioid use separately for patients who received only LMBB and those who underwent both LMBB and RFA.

## Results

**Figure 2:**
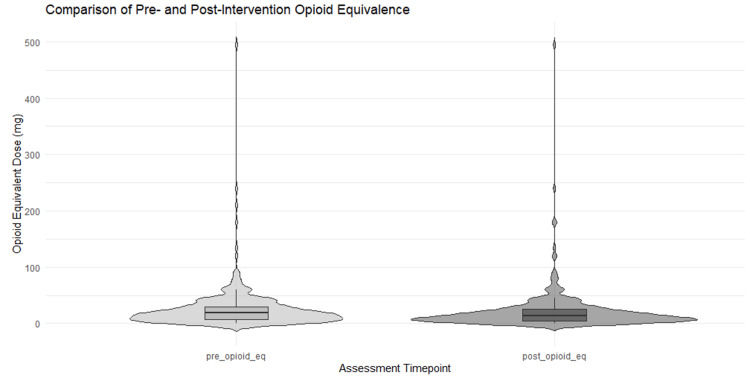
Violin plot of opioid equivalence pre- and post-intervention This figure displays the distribution of opioid equivalence (in standardized units) before (pre_opioid_eq) and after (post_opioid_eq) the intervention. Each violin plot illustrates the full distribution of values, including a box plot denoting the median and interquartile range, overlaid with individual data points.

Results can be reported as two groups' categories of data: whether our patient received just an LMBB or if the patient received a combination of the LMBB and subsequent RFA. A paired t-test was used to compare opioid equivalence doses before and after treatment in all patients who received an LMBB procedure (N = 328). The results indicated a significant reduction in post-treatment opioid equivalence compared to pre-treatment levels, t (327) = -4.65, p < 0.001. The mean difference in opioid equivalence was -4.06 (95% CI: -5.77, -2.34), suggesting a statistically significant decrease in opioid use following the intervention. To further explore the effect of RFA in addition to LMBB, a subgroup analysis was performed. Among patients who explicitly received both interventions (N = 176), a paired t-test revealed a greater reduction in opioid equivalence after treatment, t (175) = -4.75, p < 0.001. The mean difference in opioid equivalence was -6.10 (95% CI: -8.64, -3.57), indicating a significant decrease in opioid use post-treatment among this subset of patients. A violin plot illustrating opioid use before and after the procedures showed changes in the median and interquartile ranges for both groups (Figure 2).

## Discussion

The results of our study provided evidence that both the LMBB and the combination of LMBB and RFA significantly reduce prescription opioid use for patients suffering from LFA. The data showed a statistically significant reduction in opioid use post-procedure for both procedure groups, with a greater reduction in opioid use when the LMBB and RFA procedures were used.

Amidst the current opioid epidemic, the significant reduction in opioid use when interventions are completed is a promising sign. As discussed in the introduction, chronic back pain, especially from LFA, can lead to an increase in opioid prescription use. These prescriptions can lead to dependence, overdose, and a variety of public health issues that affect Americans daily.

Previous studies have shown the efficacy of pain reduction when a patient undergoes an LMBB or RFA. When an LMBB is administered without a subsequent RFA procedure, it is generally not considered therapeutic; however, it has been shown to reduce pain by 56% compared to placebo at three-month intervals [20]. When an LMBB and RFA are completed together, patients had an average pain reduction of 76.6% and an average of 30.7 weeks (about seven months) of pain relief from initial treatment, with an 83% improvement of functional status from initial treatment [21]. Even when different radiofrequency techniques, such as thermal, pulse, and cooled, were tested, researchers found significant improvement in lower facet joint pain for up to 12 months for each technique [8]. These reductions in chronic pain help to explain the eventual reduction in morphine equivalent dose represented by our collected data. With a reduction of pain, a completed LMBB/RFA can also help reduce the cost of visits to a facility. The facility price for an RFA was $186.64 and another $56.93 for every additional joint ablation, which is a one-time payment compared to the continued costs of visits to an outpatient clinic for medication management and continued injections [14]. The one-time procedure fee compared to continued healthcare visit costs helps represent the positive benefit that LMBBs and RFAs can have in the reduction of healthcare costs and overall visits to outpatient clinics.

Interventional procedures have been proven to reduce the need for pharmacological management and opioid misuse [21]. Our results align with previous studies, supporting the effectiveness of LMBBs and RFAs in reducing opioid reliance among patients with chronic back pain [13]. The substantial difference between opioid reductions and the LMBB or LMBB and RFA treatment groups emphasizes the importance of completing both procedures in sequence. Our results suggest that an LMBB alone does provide pain relief and a decrease in opioid use, but when combined with an RFA, an enhanced outcome is produced due to the thermal ablation of the nerve fibers conducting the pain. The LMBB is usually conducted as a therapeutic procedure to highlight which patients are eligible for an RFA, which is supported by the outcomes of our study, as the combination therapy led to greater reductions in opioid use and better functional outcomes. Current research outlines the added benefit of completing both the LMBB and RFA to have a more substantial reduction in pain and opioid use, which is further supported by our study [21]. The combined treatment of both the LMBB and RFA is shown to prolong pain relief by targeting the nerve fibers responsible for transmitting pain signals [22]. This combination therapy likely provides both short-term and long-term benefits, thus helping in the reduction of opioid prescriptions for longer periods, when compared to the patients who received the LMBB alone.

It is also important to acknowledge the limitations of the study. As a retrospective study, the data rely on the accuracy of patients' records and data. These data may be subject to selection bias, as the patients who received interventional procedures are more likely to have severe pain. While opioid reduction post-treatment is statistically significant, the study does not address the long-term sustainability of opioid reduction after the follow-up period. Further longitudinal studies that address the long-term impact of LMBBs or LMBB and RFA combination treatments are necessary to investigate opioid use and pain management in patients with LFA.

Our study can help contribute to the growing academic literature supporting the use of LMBBs and RFAs as effective pain management procedures and useful interventions to reduce prescription opioid use in patients suffering from LFA. These treatments offered promising solutions to reducing chronic pain from LFAs, which can help reduce the economic and societal burden caused by opioid misuse and dependence. The current strain and associated cost on the healthcare system related to pain management, procedures, and long-term treatments is significant. The opioid epidemic has further exacerbated these financial costs, where, in 2013, opioid dependence, abuse, and overdose cost the U.S. economy an estimated $78.5 billion. This amount soared to $504 billion in 2015 when accounting for illicit opioid use and overdose fatalities [19]. These findings highlight the importance of a multifaceted approach to pain management. Instead of relying on opioids and medicated pain relief, there should be a priority on interventional treatments that reduce pain, improve patient outcomes, and ultimately reduce the public health burden caused by prescription opioid use.

## Conclusions

We observed that when patients received an LMBB or a combination of an LMBB and RFA procedures, there was a decrease in prescription opioid use post-procedure. The reduction in opioid use was statistically significant when patients had just received the LMBB; however, when patients received both the diagnostic LMBB and RFA, a larger reduction in opioid use was discovered, further highlighting the importance of completing both the diagnostic LMBB and eventual RFA for maximum effectiveness of the procedure. These treatments offer promising solutions to reducing chronic pain and opioid prescription use from LFAs, with the ultimate goal of decreasing opioid misuse and dependence and the associated healthcare burden that reliance on medicated pain relief can cause. A multifaceted approach to pain management, focused more on interventional procedures and approaches, should be further investigated to reduce pain, improve patient outcomes, and reduce opioid prescription reliance and the associated public health burden.
